# Presumed ocular histoplasmosis syndrome in a commercially insured population, United States

**DOI:** 10.1371/journal.pone.0230305

**Published:** 2020-03-13

**Authors:** Kaitlin Benedict, Jessica G. Shantha, Steven Yeh, Karlyn D. Beer, Brendan R. Jackson

**Affiliations:** 1 Mycotic Diseases Branch, Division of Foodborne, Waterborne and Environmental Diseases, Centers for Disease Control and Prevention, Atlanta, Georgia, United States of America; 2 Department of Ophthalmology, Emory Eye Center, Emory University School of Medicine, Atlanta, Georgia, United States of America; University Hospitals Cleveland, UNITED STATES

## Abstract

**Purpose:**

To describe epidemiologic features of patients with presumed ocular histoplasmosis syndrome (POHS) in the United States using insurance claims data and compare POHS patients with and without choroidal neovascularization (CNV).

**Design:**

Retrospective cohort study.

**Methods:**

Patients with International Classification of Diseases, Ninth Revision, Clinical Modification diagnosis codes for histoplasmosis retinitis on an outpatient claim in the 2014 IBM^®^ MarketScan^®^ Commercial Database and the Medicare Supplemental Database who were enrolled for at least 2 years after the POHS code.

**Main outcome measures:**

Data related to testing, treatment, and direct medical costs.

**Results:**

Among >50 million total MarketScan enrollees, 6,678 (13 per 100,000) had a POHS diagnosis code. Of those, 2,718 were enrolled for 2 years; 698 (25%) of whom had a CNV code. Eleven of the 13 states with the highest POHS rates bordered the Mississippi and Ohio rivers. CNV patients had significantly more eye care provider visits (mean 8.8 vs. 3.2, p<0.0001), more ophthalmic imaging tests, higher rates of treatment with anti-vascular endothelial growth factor injections (45% vs. 4%, p<0.0001), and incurred higher mean total yearly costs ($1,251.83 vs. $251.36, p<0.0001) than POHS patients without CNV.

**Conclusions:**

Although the relationship between *Histoplasma* and POHS remains controversial, geographic patterns of POHS patient residence were consistent with the traditionally reported range of the fungus. CNV in the context of POHS was associated with additional healthcare use and costs. Further research to understand POHS etiology, risk factors, prevalence, and complications is needed, along with early diagnosis and treatment strategies.

## Introduction

*Histoplasma* spp. are environmental fungi capable of causing a wide range of human illnesses following inhalation. Most infections are asymptomatic or go unrecognized, and a smaller proportion of people develop acute or chronic pulmonary infection, disseminated disease, or other infectious or inflammatory sequelae.[1] Presumed ocular histoplasmosis syndrome (POHS) is a vision-threatening condition believed to be a long-term complication of *Histoplasma* infection; however, the link between *Histoplasma* and POHS remains somewhat controversial. Evidence supporting this relationship includes experimental infections of non-human primates, positive associations between POHS and histoplasmin skin test positivity, and the presence of POHS cases in areas where *Histoplasma* is known to be endemic.[2–6] In contrast, POHS has also been described in Europe where *Histoplasma* is not believed to be common,[7–9] although the fungus is more widespread than is currently appreciated,[10] and it is also possible that a different organism could be causing a similar syndrome in those areas. *Histoplasma* has never been cultured from a POHS-affected eye, although POHS is thought to represent a complication of infection rather than active infection. Despite the uncertainty surrounding its etiology and pathogenesis, POHS is a well-recognized entity characterized by “punched-out” round chorioretinal scars, peripapillary atrophy, the absence of vitritis, and risk of choroidal neovascularization (CNV). For some persons, these lesions do not impair vision and only require routine monitoring, whereas CNV can lead to vision loss, requiring treatment with intravitreal anti-vascular endothelial growth factor (anti-VEGF) injections or photodynamic therapy (PDT).

POHS has received little attention from a public health perspective in recent years. Given the large population residing in highly histoplasmosis-endemic areas in the United States, POHS could potentially affect hundreds of thousands of people. Furthermore, little is known about non-geographical risk factors, vision morbidity, and medical costs associated with developing CNV secondary to POHS. Here, we describe features of POHS patients with and without CNV in a large database of patients with commercial insurance, including testing, treatment, and associated direct medical costs. We hope that an updated epidemiologic description of POHS will help to increase awareness about histoplasmosis and POHS, with the ultimate goal of identifying and implementing strategies for early diagnosis and prevention.

## Methods

We used the 2014–2016 IBM^®^ MarketScan^®^ Commercial Database and the Medicare Supplemental Database, which contain individual-level health insurance claims and enrollment data for people with employer-sponsored insurance and their dependents throughout the United States. The MarketScan Research Databases are fully de-identified, so this analysis was not subject to review by the Centers for Disease Control and Prevention (CDC) institutional review board.

We used International Classification of Diseases, Ninth Revision, Clinical Modification (ICD-9-CM) diagnosis codes 115.02, 115.12, and 115.92 to identify patients with at least one outpatient claim for histoplasmosis retinitis in 2014. The index date was the date this code was first used in 2014, and we limited the cohort to patients who were continuously enrolled for 2 years after the index date. Here, we use the term POHS to refer to histoplasmosis retinitis codes, because it is more widely used in the literature. To identify features related to POHS diagnosis, treatment, and outcomes in the 2-year study window, we used ICD-9-CM, ICD-10-CM, and Current Procedural Terminology (CPT) codes (S1 Table). We calculated average patient and insurer costs associated with specific treatments for POHS. We used the Medical Care Consumer Price Index from the Bureau of Labor Statistics, US Department of Labor, to adjust costs to 2017 US Dollars.

We performed descriptive analyses and examined differences between patients with and without CNV using χ2 or Fisher exact tests for categorical variables and t-tests or Wilcoxon rank-sum tests for continuous variables. To contextualize the prevalence of POHS in the MarketScan population, we also determined the number of patients in 2014 with ICD-9-CM codes on outpatient claims for any type of histoplasmosis (115.x) as well as certain other causes of eye infection: herpetic eye disease (054.40, 054.42, 054.43, 054.44, and 054.49), toxoplasmosis retinitis (130.2), and syphilis retinitis (090.0, 090.3, 090.40, 090.49, 090.5, 091.5x, 094.3, 094.83, 094.85, 094.89, and 094.9).

## Results

**Fig 1:**
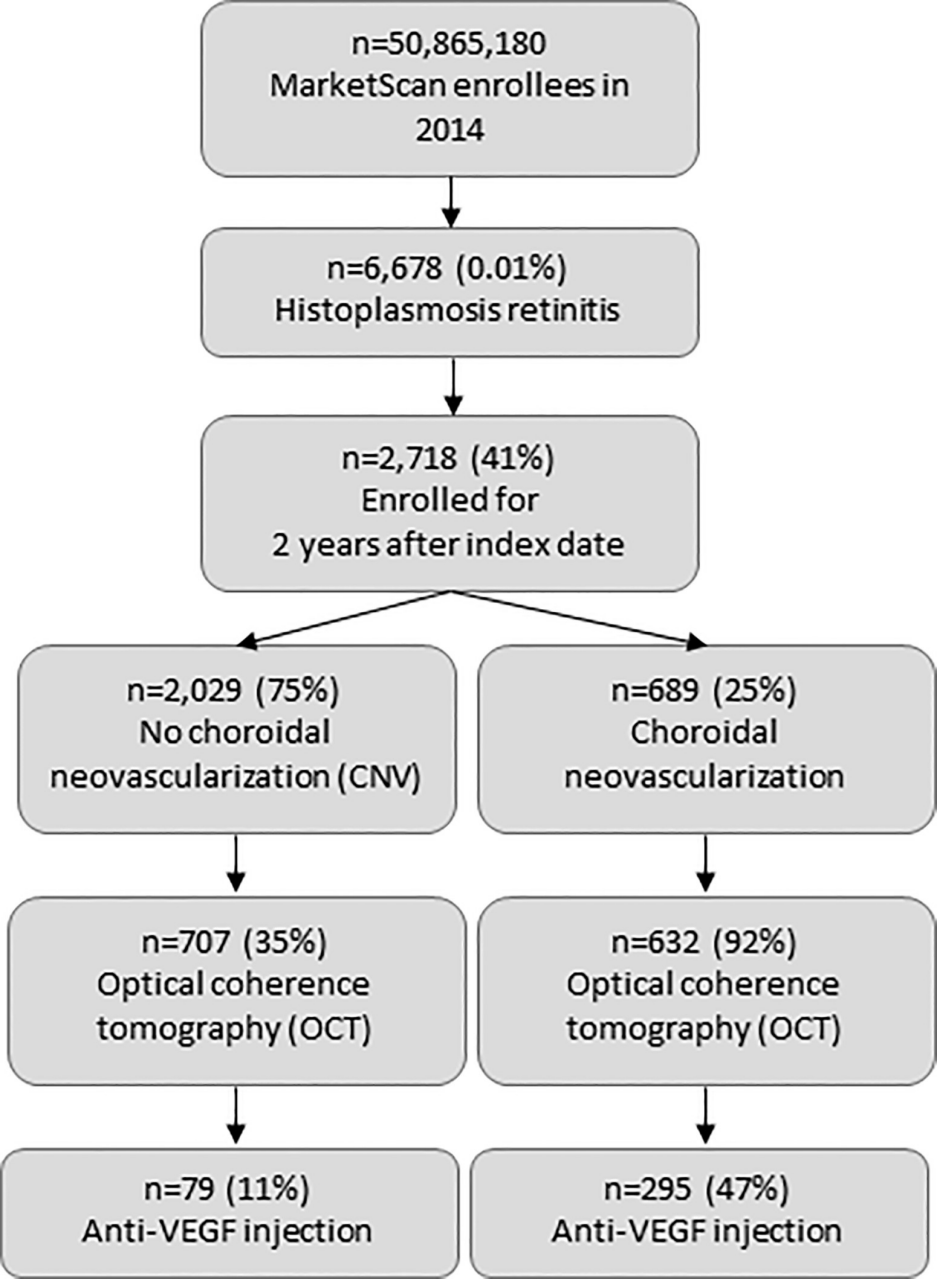
Enrollment and treatment, patients with presumed ocular histoplasmosis syndrome, 2014.

Of more than 50 million total MarketScan enrollees in 2014, 6,678 had an ICD-9-CM code for histoplasmosis retinitis on an outpatient claim (POHS patients), compared with 15,122 for herpetic eye disease, 1,499 for syphilis retinitis, and 1,326 for toxoplasmosis retinitis. Among patients with any histoplasmosis ICD-9-CM code (115.x), 69% had histoplasmosis retinitis codes, and 9% had a code for “unspecified histoplasmosis” assigned by an eye care provider. Of the 6,678 POHS patients, 2,718 were enrolled for 2 years after the index date (Fig 1). Of those, 698 (25%) had at least one diagnosis code for CNV, and 2,029 (75%) did not.

**Fig 2:**
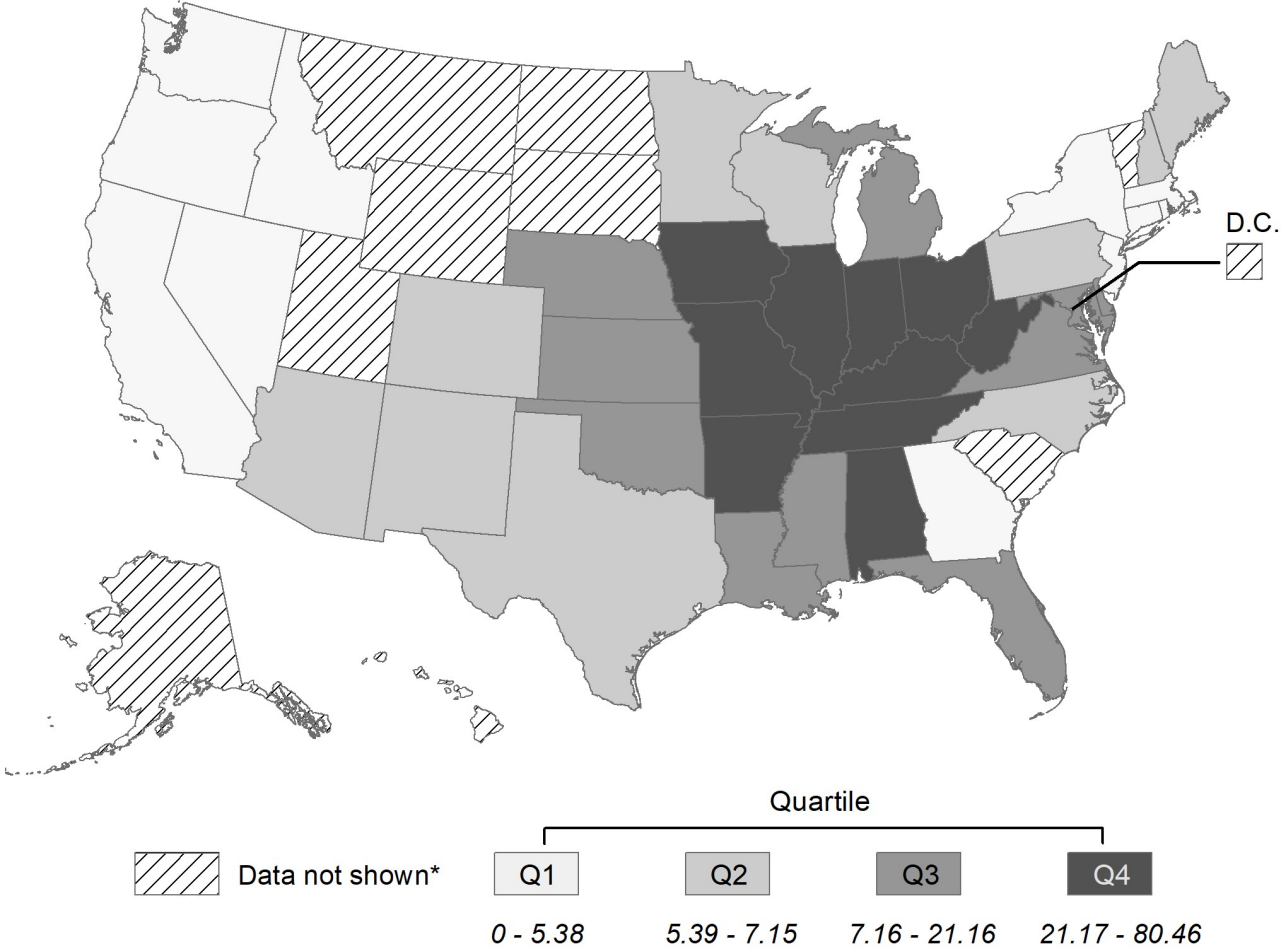
Rates of presumed ocular histoplasmosis syndrome per 100,000 MarketScan enrollees, 2014. *To avoid unreliable estimates, rates not calculated for states with <5 cases. Reporting MarketScan data from South Carolina is not permitted.

CNV patients were significantly younger (mean 53.4 vs. 58.2 years, p<0.0001) and more likely to be male (39% vs. 35%, p<0.0482) than those without CNV (Table 1). Half (50%) of POHS patients lived in the South census region, and 41% lived in the Midwest (Fig 2). POHS rates (including patients not enrolled for the entire study window) among MarketScan enrollees were highest in the lower Midwest and South Central regions, with lower rates in surrounding states and very low rates in much of New England and the West. In contrast, rates of herpetic eye disease, syphilis retinitis, and toxoplasmosis retinitis clustered less by region (S1–S3 Figs).

**Table 1:** Demographic features and specific diagnoses or procedures among patients with presumed ocular histoplasmosis syndrome, 2014.

Demographics at index date	ALL PATIENTS		NO CNV		CNV		p-value
	n = 2718	%	n = 2029	%	n = 689	%	
Age in years, median, mean (range)	58.0, 57.0	1–95	59.0, 58.2	1–95	55.0, 53.4	15–95	<0.0001
Age group in years							<0.0001
0–17	24	1%	21	1%	3	0%	
18–34	162	6%	100	5%	62	9%	
35–44	319	12%	194	10%	125	18%	
45–54	552	20%	402	20%	150	22%	
55–64	921	34%	701	35%	220	32%	
65 and older	740	27%	611	30%	129	19%	
Sex							0.0482
Male	988	36%	716	35%	272	39%	
Female	1730	64%	1313	65%	417	61%	
Census division of primary beneficiary’s residence							0.4741
New England	18	1%	14	1%	4	1%	
Mid-Atlantic	118	4%	83	4%	34	5%	
East North Central	887	33%	667	33%	220	32%	
West North Central	217	8%	154	8%	63	9%	
South Atlantic	355	13%	257	13%	99	14%	
East South Central	760	28%	576	28%	183	27%	
West South Central	283	10%	222	11%	61	9%	
Mountain	28	1%	19	1%	10	1%	
Pacific	51	2%	36	2%	15	2%	
Unknown	1	0%	1	0%	0	0%	
**Other diagnoses or procedures in the 2-year study window**							
Disseminated histoplasmosis	17	1%	13	1%	4	1%	1.0000
Unspecified or other forms of histoplasmosis	919	34%	521	26%	398	58%	<0.0001
Pulmonary histoplasmosis	12	0%	6	0%	6	1%	0.0871
Histoplasmosis antibody or antigen test	28	1%	14	1%	14	2%	0.0026
Fungal culture or smear	127	5%	98	5%	29	4%	0.5046
Chorioretinitis	74	3%	49	2%	25	4%	0.0908
Vitreous disorders	720	26%	495	24%	225	33%	<0.0001
Diabetic retinopathy	86	3%	66	3%	20	3%	0.6501
Diabetic macular edema	24	1%	18	1%	6	1%	0.9685
Macular degeneration (AMD)	652	24%	410	20%	242	35%	<0.0001
History of or current tobacco use	312	11%	232	11%	80	12%	0.8999

Macular degeneration was more frequently coded for CNV patients than for non-CNV patients (35% vs. 20%,), as were vitreous disorders (33% vs. 24%) (p<0.0001 for both). Eleven percent (n = 312) had diagnosis codes for a history of or current tobacco use, but there was no difference between CNV and non-CNV patients.

Most patients (86%, n = 2,326) visited an eye care provider on the index date, and nearly half (49%, n = 1,320) had a CPT code for a routine eye examination on the index date (52% of non-CNV patients vs. 38% of CNV patients, p<0.0001) (Table 2). CNV patients had significantly more visits (mean 8.8, range 0–46) to an eye care provider during the study window than non-CNV patients (mean 3.2, range 0–42) (P<0.0001). Nearly all (92%) CNV patients had CPT codes for optical coherence tomography (OCT), vs. approximately one-third (35%) of non-CNV patients (p<0.0001), and 79% of CNV patients underwent OCT ≥3 times during the 2-year follow up period. CNV patients were also more likely to undergo fluorescein angiography (38%) than were non-CNV patients (8%) (p<0.0001).

**Table 2:** Diagnosis, treatment, and outcomes among patients with presumed ocular histoplasmosis syndrome, 2014.

	ALL PATIENTS		NO CNV		CNV		p-value
	n = 2718	%	n = 2029	%	n = 689	%	
Visited an eye care provider on the index date	2326	86%	1716	85%	610	89%	0.0106
Routine eye examination during study window	1871	69%	1386	68%	485	70%	0.3079
Routine eye examination on the index date	1320	49%	1061	52%	259	38%	<0.0001
Mean, median number of visits to an eye care provider (range)	4.7, 3.0	(0–46)	3.2, 2.0	(0–42)	8.8, 7.0	(0–46)	<0.0001
0 visits	304	11%	250	12%	54	8%	
1 visit	449	17%	422	21%	27	4%	
2 visits	559	21%	508	25%	51	7%	
3 or more visits	1406	52%	849	42%	557	81%	
Intravitreal injection	565	21%	113	6%	452	66%	<0.0001
Subtenon injection	10	0%	3	0%	7	1%	0.0038
Steroid injection	325	12%	248	12%	77	11%	0.4642
Oral steroids (n = 2576)	845	33%	635	33%	210	32%	0.5740
Any anti-VEGF injection	391	14%	84	4%	307	45%	<0.0001
Mean, median number of injections (range)	7.1, 5.0	(1–44)	(8.0, 5.0)	1–44	(6.9, 4.0)	1–43	0.2996
Aflibercept	69	3%	27	1%	42	6%	<0.0001
Ranibizumab	30	1%	20	1%	40	6%	<0.0001
Bevacizumab	305	11%	48	2%	257	37%	<0.0001
Fluorescein angiography	420	15%	159	8%	261	38%	<0.0001
Once	114	27%	41	26%	73	28%	
Twice	182	43%	88	55%	94	36%	
Three or more times	124	30%	30	19%	94	36%	
Photodynamic therapy	37	1%	4	0%	33	5%	<0.0001
Once	5	14%	2	25%	3	9%	
Twice	21	57%	2	25%	19	58%	
Three or more times	11	30%	0	0%	11	33%	
Optical coherence tomography	1339	49%	707	35%	632	92%	<0.0001
Once	354	26%	295	42%	59	9%	
Twice	230	17%	159	22%	71	11%	
Three or more times	755	56%	253	36%	502	79%	
Fundus photography	1114	41%	791	39%	323	47%	0.0003
Once	610	55%	448	57%	162	50%	
Twice	320	29%	239	30%	81	25%	
Three or more times	184	17%	104	13%	80	25%	
Vision loss	57	2%	35	2%	22	3%	0.0201

Two-thirds (66%) of CNV patients received an intravitreal injection with any agent (e.g., anti-VEGF agents, corticosteroids) compared with 6% of non-CNV patients (p<0.0001). Forty-five percent (n = 307) of CNV patients received at least one anti-VEGF injection, with a mean of 6.9 injections (range 1–43) during the 2-year study window; 37% received bevacizumab, 6% received aflibercept, and 6% received ranibizumab. Five percent of CNV patients received photodynamic therapy vs. <1% of non-CNV patients. CNV and non-CNV patients were equally likely to receive corticosteroid injections (11% vs. 12%) or oral corticosteroids (32% vs. 33%). Diagnosis codes for vision loss were infrequent but more common for patients with CNV than for those without (3% vs. 2%, p = 0.02).

The mean total yearly patient out-of-pocket cost for POHS-related visits was $64.32 for non-CNV patients ($187.04 to the insurer) and $248.19 for CNV patients ($1,003.64 to the insurer) (p<0.0001) (Table 3). The mean costs of an anti-VEGF injection were $50.98 to patients and $884.67 to the insurer, with mean total cost being highest for aflibercept ($2,256.17) and lowest for bevacizumab ($122.07). Mean total cost of photodynamic therapy was also substantial ($891.14).

**Table 3:** Mean costs of visits and treatments related to presumed ocular histoplasmosis syndrome, 2014.

Mean costs	ALL PATIENTS	NO CNV	CNV	p-value
Cost of POHS visit to patient	80.58	68.63	91.91	<0.0001
Cost of POHS visit to insurer	288.26	200.33	371.63	<0.0001
Cost of intravitreal injection to patient	39.14	22.99	42.86	<0.0001
Cost of intravitreal injection to insurer	169.05	143.82	174.87	<0.0001
Cost of anti-VEGF injection to patient	50.98	66.62	46.02	0.0094
Cost of anti-VEGF injection to insurer	884.67	1295.48	754.47	<0.0001
Cost of aflibercept to patient	97.04	80.77	106.11	0.1331
Cost of aflibercept to insurer	2159.13	2124.92	2178.20	0.5125
Cost of ranibizumab to patient	130.52	162.04	115.25	0.1028
Cost of ranibizumab to insurer	2036.77	1955.84	2075.96	0.1059
Cost of bevacizumab to patient	12.46	6.95	13.63	<0.0001
Cost of bevacizumab to insurer	109.61	347.45	59.16	<0.0001
Cost of photodynamic therapy to patient	185.23	529.80	164.76	0.3645
Cost of photodynamic therapy to insurer	705.91	711.36	705.59	0.9873
Total yearly cost of POHS visits to patient	113.32	64.32	248.19	<0.0001
Total yearly cost of POHS visits to insurer	404.64	187.04	1003.64	<0.0001

To assess the potential contribution of age-related macular degeneration (AMD) to treatments and outcomes among POHS patients, we examined the POHS cohort after excluding the 652 patients who had AMD codes. Compared with the full cohort, the remaining 2,066 POHS patients without AMD were younger (median 56 vs. 58 years) and less frequently had CNV codes (22% vs. 26%), any intravitreal injection (14% vs. 21%), and any anti-VEGF injection (9% vs. 14%). The mean total yearly patient out-of-pocket cost for POHS-related visits was $97.68 for those without AMD vs. $113.32 for the full cohort, and total yearly cost to insurer was $259.08 vs. $404.64.

## Discussion

We describe a large cohort of patients with diagnosis codes for POHS in the United States using medical claims data from a commercially-insured population. Our results show that the prevalence of POHS is approximately 13 cases per 100,000 MarketScan enrollees, with a geographic pattern consistent with the traditionally described distribution of *Histoplasma*, concentrated around the Ohio and Mississippi River Valleys. Remarkably, it accounted for over two-thirds of overall histoplasmosis claims of any type, including the pulmonary form thought to be much more common. In accordance with the known predisposition for POHS to cause asymptomatic scarring in some people and devastating vision loss in others, in this cohort, CNV was associated with certain demographic features, greater healthcare usage, and higher costs, which has implications from clinical, health economics, and public health perspectives.

POHS is often described in scientific literature as a “leading cause of vision loss” among young adults in the United States, though the precise origin of this statement is unclear. Despite the fact that MarketScan databases are not directly representative of the national population and that we were unable to calculate prevalence rates in the continuously-enrolled POHS patient group, POHS diagnoses were not uncommon based solely on the number of cases, particularly compared with other infectious causes of retinitis such as syphilis and toxoplasmosis.

Much of the information about the estimated prevalence of POHS and its complications is half a century old. Studies performed in the 1960s and 70s identified rates of POHS in the general community ranging from 1.6% in Ohio [2] to 2.7% in Maryland, with a slightly higher prevalence (4.4%) among persons with positive histoplasmin skin tests.[11] Large-scale histoplasmin skin tests surveys conducted in the 1940s and 50s identified nine states (Alabama, Arkansas, Illinois, Indiana, Iowa, Kentucky, Missouri, Ohio, Tennessee) with large areas of positivity rates >70%.[12] Applying this proportion to the nearly 60 million people currently living in those areas, we estimate that 42 million people would have positive histoplasmin skin tests (although the test is now unavailable), and of those, 672,000 would have ocular findings consistent with POHS in those 9 states alone, using the most conservative estimate of POHS prevalence of 1.6%. In our analysis, up to a quarter of patients with POHS diagnosis codes also had CNV coded and therefore likely have some degree of vision loss. It is unclear to what extent these patients represent the broader population with POHS, but regardless they suggest that POHS-induced vision defects may be relatively common. Although vision loss is difficult to ascertain with administrative data and appears to be substantially under-coded, most patients with CNV likely experience some degree of vision impairment. Additional research to understand the current prevalence of POHS and associated vision loss would help to understand its importance in the context of other major causes of vision loss and impairment.

A fundamental question about POHS is whether it is truly caused by *Histoplasma*. In this analysis, the geographic distribution of POHS patients was remarkably similar to that of positive histoplasmin skin test results,[12] which could support the conclusion that *Histoplasma* infection is a causative component of POHS. This pattern could be influenced by greater provider awareness of POHS in known endemic areas, but it is unlikely to be completely explained by this phenomenon alone. In general, histoplasmosis is an under-recognized infection in the United States,[13] and information about its long-term sequelae, including POHS, is limited. We hope that that future experimental and epidemiologic studies can further clarify the relationship between *Histoplasma* and POHS. One study provided promising evidence by detecting *H*. *capsulatum* DNA in a patient’s chronic choroidal lesions.[14]

In this cohort, one-quarter of patients had CNV diagnosis codes, which is higher than the estimate that fewer than 5% of people with POHS develop CNV.[15] The high proportion with CNV seen here could represent selection bias towards patients who are more likely to be symptomatic and therefore seek care. An alternative explanation for the high prevalence of CNV could be related to misclassification with AMD, which also appeared to be common. Although it seems unlikely that patients would have both AMD and POHS, clinical phenotypic similarities including retinal pigment epithelium atrophy and pigment change may be observed in both conditions, leading to use of both diagnostic codes. The young age of patients with POHS and CNV suggests POHS as the predisposing disease entity versus AMD. Additionally, although CNV and intravitreal injections were less common among POHS patients without AMD codes than those with AMD codes, nearly a quarter of non-AMD POHS patients had CNV and one in seven received intravitreal injections, suggesting that POHS is independently associated with CNV, apart from AMD.

Another notable demographic finding is the higher proportion of POHS in females, whereas previous studies have shown that POHS appears to affect males and females similarly [11] or have a slight predominance in males, similar to histoplasmosis in general.[6, 13] These differences could be related to the convenience sample nature of MarketScan data or to differences in care-seeking behavior by sex. In contrast to prior reports, we did not find an association between smoking and CNV.[16] This could be due to under-coding of smoking status in administrative data or to our use of POHS patients without CNV as the comparison group, if smoking increases the risk for POHS itself. This explanation seems plausible given the higher rate of previous or current tobacco use in POHS patients (11%) compared with the general MarketScan population (7%).

The pattern of retinal imaging for initial diagnosis and follow-up monitoring was notable. In this cohort, patients with CNV were more likely to undergo OCT and FA testing than non-CNV patients, consistent with the need for routine monitoring in active CNV, including follow-up of treatment with anti-VEGF injection and PDT. Together, this monitoring and treatment was responsible for the substantially greater cost to the patient and insurer of CNV.

Our finding that over half of non-CNV POHS patients had a routine eye examination on the index date supports the notion that many POHS cases are discovered incidentally and might only require routine observation to monitor for the development of CNV. Requiring a specific period of time without the diagnosis of interest (i.e., POHS) before the index date is a common method in claims-based research to attempt to identify incident cases. However, establishing such a time window is difficult for long-term conditions such as POHS; therefore, our cohort includes both incident and prevalent cases. Among patients with POHS diagnosis codes in 2014, 28%, 50%, and 55% of patients also had POHS diagnosis codes in the 1 year, 2, years, and 3 years before the index date, respectively, confirming disease monitoring over several years. Consequently, CNV is likely over-represented in our cohort, in addition to previously mentioned reasons related to potential misclassification of macular degeneration and selection bias.

Formal treatment guidelines for POHS do not currently exist. Antifungal therapy is not indicated because POHS is not believed to represent an active infectious process.[17] A common approach to managing POHS patients without CNV includes patient education about the risk of developing CNV and the importance of self-monitoring for vision changes. Treatment of CNV is often successful with administration of anti-VEGF agents.[18, 19] Our findings are consistent with these management patterns, with most of the anti-VEGF agent use occurring in the CNV group. PDT and corticosteroid use were also observed although with lower frequency compared with anti-VEGF therapy. Moreover, our estimates of OCT frequency are consistent with OCT-guided personalized treatment for CNV described in the literature and likely reflect current clinical practice. However, the treatment algorithms (i.e., treat-and-extend vs. treatment pro re nata [PRN]) could not be ascertained from this dataset.

Total yearly costs associated with POHS were substantial, exceeding $500 per patient, though we were not able to approximate total costs nationwide. The overall economic burden of major eye disorders in the US is estimated to be more than $35 billion among adults >40 years alone, only 46% of which is attributable to direct medical costs.[20] Our cost estimates do not capture direct nonmedical costs or indirect costs, which are likely to be substantial for POHS patients with CNV-associated vision impairment, as has been previously shown for patients with AMD.[21] Specifically, we found that the costs associated with anti-VEGF treatments were high, with some patients receiving many injections during the study window. A recent European study concluded that bevacizumab was more cost-effective than ranibizumab or aflibercept for treatment of AMD.[22] Although we were not able to evaluate cost-effectiveness, bevacizumab was used more commonly than other anti-VEGF agents in our study, likely due to its lower cost and previously described efficacy.[23]

In addition to the limited information available about the overall magnitude of POHS as a cause of vision loss in the United States, few published data exist regarding its personal burden on patients, though a small qualitative study suggests that POHS patients can experience substantial emotional distress and impaired productivity due to vision loss and treatment-related side effects.[24] Greater awareness about POHS among the medical community and increased collaborations across specialties and disciplines (i.e., ophthalmologists, general practitioners, infectious disease specialists, and public health professionals) could be helpful to better characterize the syndrome and its effects on patients.

Strengths of this study include the large sample size and the ability to examine diagnosis and procedure codes over a 2-year period. The limitations of using administrative data for epidemiologic studies are well-recognized and have been previously reviewed specifically in the context of ophthalmology research.[25] Misclassification due to unintentional miscoding or coding practices that influence reimbursement is a particularly important consideration in analyses of billing data [26]. For example, patients may be assigned AMD diagnosis codes to obtain reimbursement for anti-VEGF injections. Evaluating clinical features such as disease severity is another common challenge with claims-based data, although the presence of a CNV code among patients with histoplasmosis retinitis codes appeared to identify more patients with more severe cases in this analysis. Unfortunately, single codes to identify “histoplasmosis retinitis” are no longer available in the ICD-10-CM coding system; instead, POHS diagnoses are coded using B39 for histoplasmosis and H32 for “chorioretinal disorders in diseases classified elsewhere.” From a public health perspective, this change could potentially make tracking POHS trends more challenging. Lastly, because these MarketScan data only represent people with private insurance, the findings might not be generalizable to uninsured people or those with government insurance plans without supplemental insurance coverage.

Our study did not address the role of early detection and treatment in preventing further complications in POHS-associated CNV, but this remains an important goal. Future research is warranted to better understand *Histoplasma* as a possible cause of POHS and risk factors for developing POHS and associated vision loss.

## Supporting information

S1 Table Diagnosis and procedure codes for features of interest related to presumed ocular histoplasmosis syndrome. (DOCX)

S1 Fig Rates of herpetic eye disease per 100,000 MarketScan enrollees, 2014. *To avoid unreliable estimates, rates not calculated for states with <5 cases. Reporting MarketScan data from South Carolina is not permitted.
(TIF)

S2 Fig Rates of syphilis retinitis per 100,000 MarketScan enrollees, 2014. *To avoid unreliable estimates, rates not calculated for states with <5 cases. Reporting MarketScan data from South Carolina is not permitted.
(TIF)

S3 Fig Rates of toxoplasmosis retinitis per 100,000 MarketScan enrollees, 2014. *To avoid unreliable estimates, rates not calculated for states with <5 cases. Reporting MarketScan data from South Carolina is not permitted.
(TIF)
