# Supplementary material for: Presumed ocular histoplasmosis syndrome in a commercially insured population, United States
Source: PLoS One. 2020 Mar 13;15(3):e0230305. doi: 10.1371/journal.pone.0230305 (PMC7069623; doi:10.1371/journal.pone.0230305)
Supplement: S1 Table — (DOCX) [file pone.0230305.s001.docx]

| **Variable** | **Diagnosis or procedure code** |
| --- | --- |
| Any histoplasmosis | ICD-9 codes 115.xx or ICD-10 code B39.x |
| Disseminated histoplasmosis | ICD-9 codes 115.01, 115.03, 115.04, 115.11, 115.13, 115.14, 115.91, 115.93, 115.94; ICD-10 code B39.3 |
| Unspecified or other forms of histoplasmosis | ICD-9 codes 115.00, 115.09, 115.10, 115.19, 115.90, 115.99; ICD-10 code B39.4, B39.5, B39.9 |
| Pulmonary histoplasmosis | ICD-9 codes 115.05, 115.15, 115.95; ICD-10 codes B39.0, B39.1, B39.2 |
| Histoplasmosis retinitis by ICD-9 code | ICD-9 codes 115.02, 115.12, 115.92 |
| Histoplasmosis retinitis by ICD-10 code | ICD-10 codes B39.x and H32 listed together on a claim |
| Choroidal neovascularization | ICD-9 code 362.16; ICD-10 code H35.05x |
| Chorioretinitis | ICD-9 code 363.0-363.2; ICD-10 code H30.x |
| Vitreous disorders | ICD-9 code 379.2x; ICD-10 codes H43.x |
| Vision loss | ICD-9 code 369.x; ICD-10 code H54.x |
| Diabetic retinopathy | ICD-9 code 362.0x; ICD-10 codes E11.3x, E08.3x, E09.3x, E10.3x, E13.3x |
| Diabetic macular edema | ICD9 code 362.07; ICD-10 codes ICD10 E11.311, E11.321x, E11.331x, E11.341x, E11.351x, E11.37x, E08.311, E08.321x, E08.331x, E08.341x, E08.351x, E08.37x, E09.311, E09.321x, E09.331x, E09.341x, E09.351x, E09.37x, E10.311, E10.321x, E10.331x, E10.341x, E10.351x, E10.37x, E13.311, E13.321x, E13.331x, E13.341x, E13.351x, E13.37x |
| Macular degeneration | ICD-9 code 362.5x; ICD-10 code H35.3x |
| History of or current tobacco use | ICD-9 codes 305.1, V15.82; ICD-10 code F17, Z87.891, Z72.0 |
| Histoplasmosis antibody or antigen test | CPT codes 86698, 86671, 87385 |
| Fungal culture or smear | CPT codes 87205, 87206, 87210, 87106, 87103, 87101, 87107, 87102 |
| Intravitreal injection of a pharmacologic agent | CPT code 67028 |
| Injection of medication or other substance into Tenon's capsule | CPT code 67515 |
| Steroid injection | CPT codes J7312, J3301, J3300 |
| Aflibercept injection | CPT codes Q2046, J0178, C9291 |
| Ranibizumab injection | CPT codes J2778, C9233 |
| Bevacizumab injection | CPT codes C9257, C9214, J9035, S0116, Q2024 |
| Anti-vascular endothelial growth factor injection | CPT codes for aflibercept, ranibizumab, or bevacizumab injection |
| Fluorescein angiography | CPT code 92235 |
| Photodynamic therapy | CPT codes 67210, J3396, 67225, 67221 |
| Optical coherence tomography | CPT code 92134 |
| Fundus photography | CPT code 92250 |
| Routine ophthalmology visit | CPT codes 92002, 92004, 92012, 92014 |
