# Supplementary figures and images for: Presumed ocular histoplasmosis syndrome in a commercially insured population, United States
Source: PLoS One. 2020 Mar 13;15(3):e0230305. doi: 10.1371/journal.pone.0230305 (PMC7069623; doi:10.1371/journal.pone.0230305)

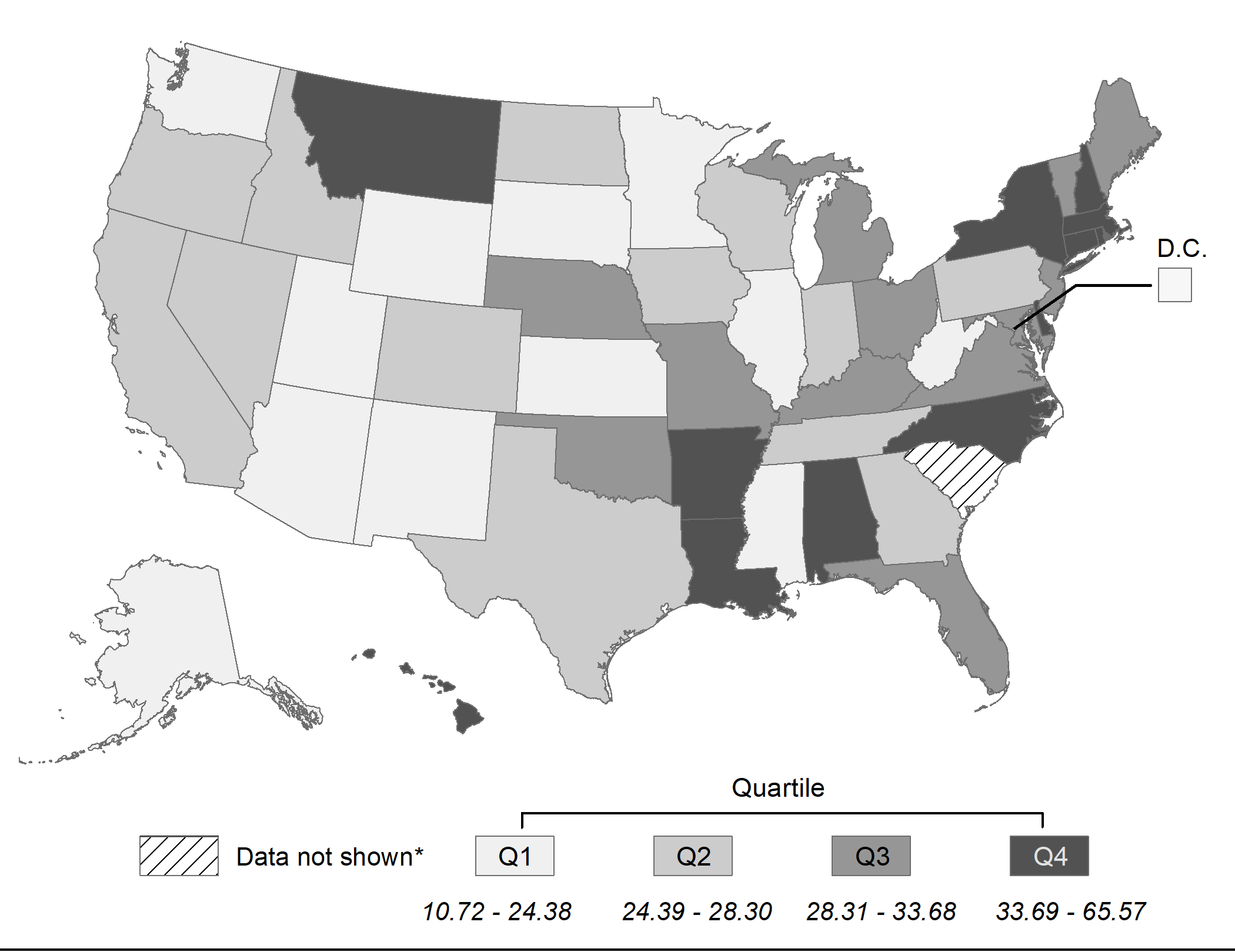

Supplement: S1 Fig — *To avoid unreliable estimates, rates not calculated for states with <5 cases. Reporting MarketScan data from South Carolina is not permitted. (TIF) [file pone.0230305.s002.tif]

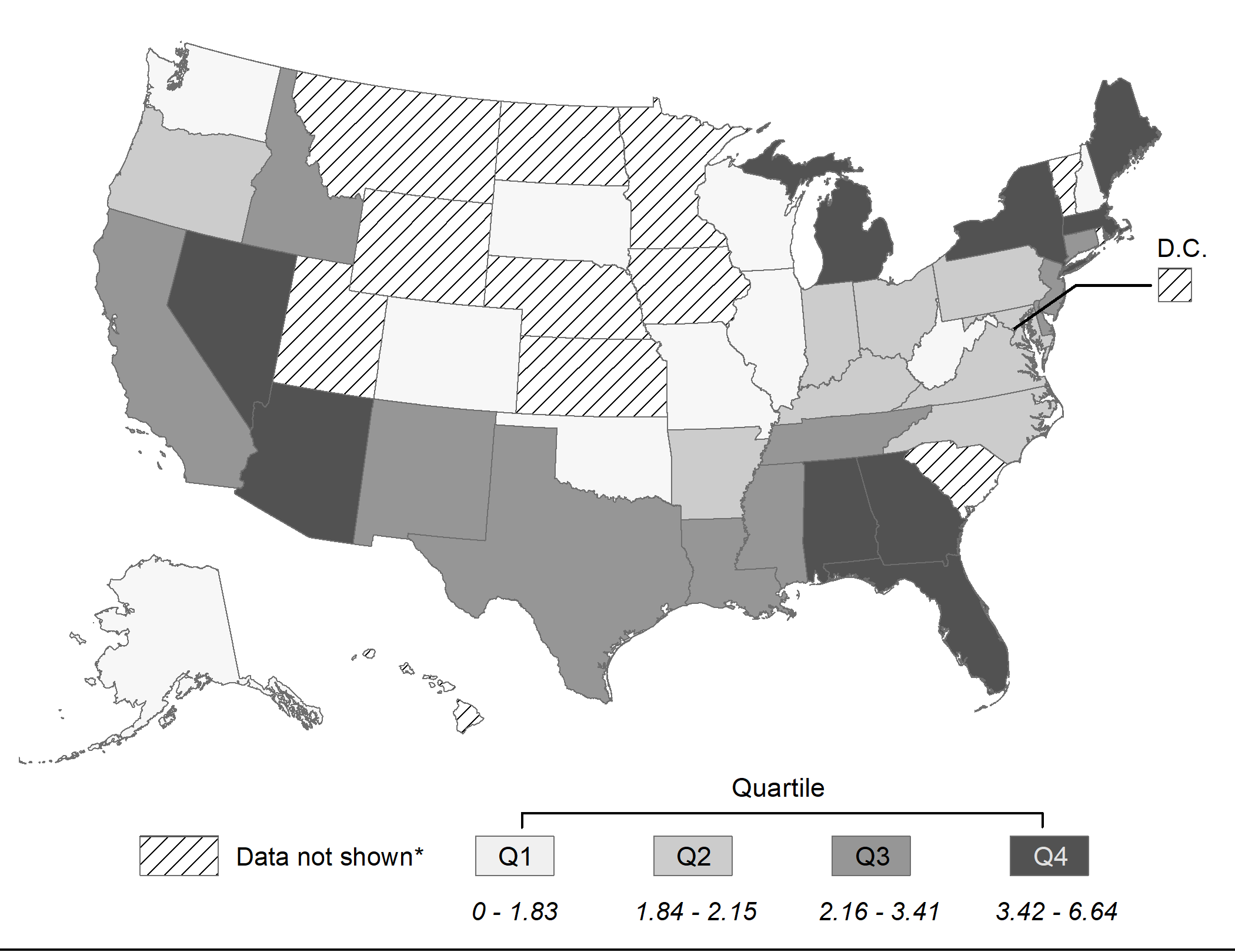

Supplement: S2 Fig — *To avoid unreliable estimates, rates not calculated for states with <5 cases. Reporting MarketScan data from South Carolina is not permitted. (TIF) [file pone.0230305.s003.tif]

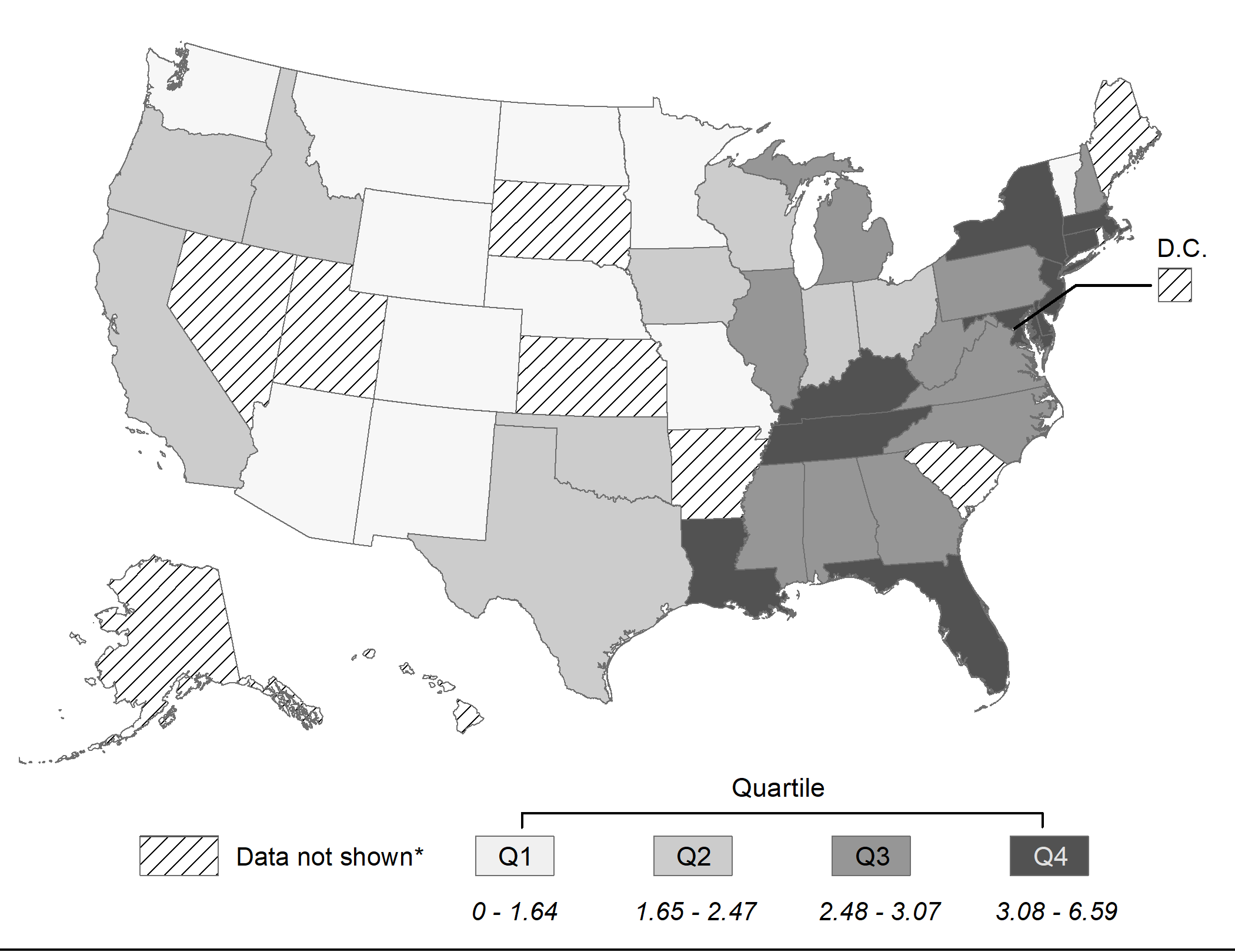

Supplement: S3 Fig — *To avoid unreliable estimates, rates not calculated for states with <5 cases. Reporting MarketScan data from South Carolina is not permitted. (TIF) [file pone.0230305.s004.tif]
